# Nanostructure-Enabled and Macromolecule-Grafted Surfaces for Biomedical Applications

**DOI:** 10.3390/mi9050243

**Published:** 2018-05-17

**Authors:** Madeline Small, Addison Faglie, Alexandra J. Craig, Martha Pieper, Vivian E. Fernand Narcisse, Pierre F. Neuenschwander, Shih-Feng Chou

**Affiliations:** 1 Department of Mechanical Engineering, College of Engineering, The University of Texas at Tyler, 3900 University Blvd., Tyler, TX 75799, USA; msmall4@patriots.uttyler.edu (M.S.); AFaglie@patriots.uttyler.edu (A.F.); ACraig4@patriots.uttyler.edu (A.J.C.); mpieper@patriots.uttyler.edu (M.P.); 2 Department of Chemistry and Physics, School of Arts and Sciences, LeTourneau University, Longview, TX 75607, USA; vivianfernandnarcisse@letu.edu; 3 Department of Cellular and Molecular Biology, The University of Texas Health Science Center at Tyler, Tyler, TX 75708, USA; Pierre.Neuenschwander@uthct.edu

**Keywords:** macromolecules, grafting, surfaces, thrombosis, wound healing

## Abstract

Advances in nanotechnology and nanomaterials have enabled the development of functional biomaterials with surface properties that reduce the rate of the device rejection in injectable and implantable biomaterials. In addition, the surface of biomaterials can be functionalized with macromolecules for stimuli-responsive purposes to improve the efficacy and effectiveness in drug release applications. Furthermore, macromolecule-grafted surfaces exhibit a hierarchical nanostructure that mimics nanotextured surfaces for the promotion of cellular responses in tissue engineering. Owing to these unique properties, this review focuses on the grafting of macromolecules on the surfaces of various biomaterials (e.g., films, fibers, hydrogels, and etc.) to create nanostructure-enabled and macromolecule-grafted surfaces for biomedical applications, such as thrombosis prevention and wound healing. The macromolecule-modified surfaces can be treated as a functional device that either passively inhibits adverse effects from injectable and implantable devices or actively delivers biological agents that are locally based on proper stimulation. In this review, several methods are discussed to enable the surface of biomaterials to be used for further grafting of macromolecules. In addition, we review surface-modified films (coatings) and fibers with respect to several biomedical applications. Our review provides a scientific update on the current achievements and future trends of nanostructure-enabled and macromolecule-grafted surfaces in biomedical applications.

## 1. Introduction

The development of biocompatible and biodegradable polymers has enabled the use of injectable and implantable biomaterials for various diseases. To successfully utilize the advantages of their functions for disease treatment, the key component of the strategy relies on the surface properties of the materials. From a materials science (or polymer science) point of view, previous efforts have been focused on the development of polymeric drug carriers and/or tissue scaffolds with bulk properties that match the surrounding tissues for a particular application. However, adverse side effects in device rejection were noticeable as a result of inflammation, abnormal tissue growth, and cytotoxicity. In addition, the conventional polymer surfaces exhibited poor biocompatibility, resulting in critical obstacles, such as undesirable protein adsorption and cell adhesion, for biomedical applications. Based on these observations, the focus of the injectable and implantable biomaterials has translated into the understanding and the design of desirable surfaces properties for the materials. Since most of the injectable and implantable biomaterials are based on polymers, the effects of surface hydrophobicity, polarity, roughness, and texture will play important roles in cellular responses. To solve these problems, numerous basic and applied works have been explored on the surface modifications of polymeric materials for biomedical applications [1,2,3].

The importance of surface properties in injectable and implantable biomaterials was addressed through surface modifications in various fields with different innovative techniques, including chemical and physical processes. Physical processes utilize techniques to induce surface segregation. For example, polymeric surfaces may be radiated by electromagnetic waves or oxidized by gases in order to create the surface features. In contrast, chemical modifications involve the use of wet-treatment, blending, coating, and etching. In this review, we provide several methods for the surface modifications of materials, in which macromolecules can be grafted onto the surface of the materials providing improved surface properties. Grafting is a process to attach molecules onto the surface of an object, and its advantages include the easy introduction, controllable density, and exact localization of graft chains at the surface, without changing the bulk properties of the materials. In some cases, graft chains via covalent bonding onto polymer surfaces are necessary to avoid molecule delamination with long-term chemical stability, which has a superior advantage over physical grafting methods.

**Figure 1:**
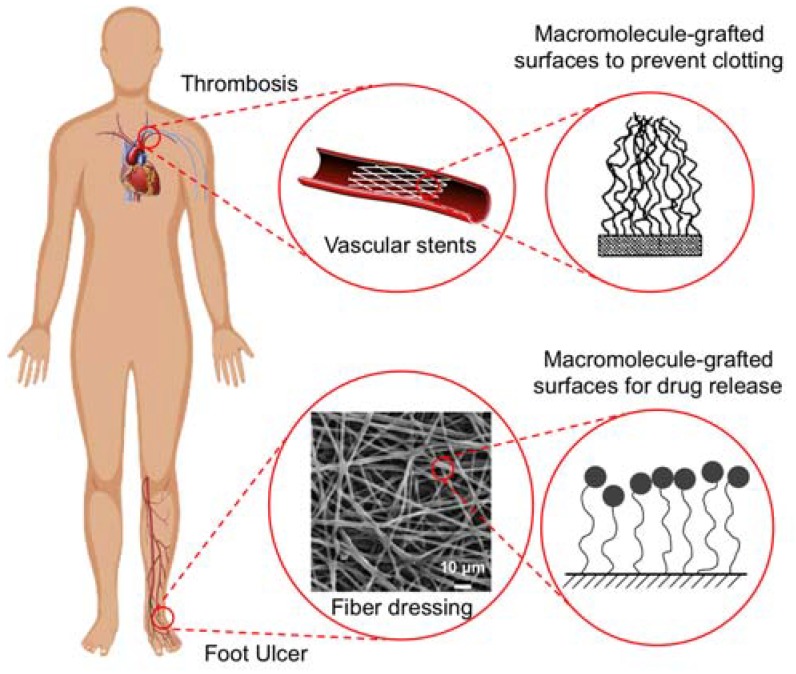
Surface functionalization of injectable and implantable biomaterials with macromolecules illustrated in biomedical applications of thrombosis and non-healing wounds. The attachment of the surface features on medical devices creates nanostructure-enabled and macromolecule-grafted surfaces that prevent blood clotting in thrombosis, as well as promoting the controlled drug delivery from nanofibrous dressing for non-healing wounds.

The primary function of the injectable and implantable biomaterials is to provide therapeutic performance locally. Surface grafting of macromolecules not only enhances the compatibility of the materials with surrounding tissues, but it also can be utilized as the module for drug delivery (Figure 1). In the former case, we emphasize the review of vascular stents and their surface coatings. This is to provide an alternative strategy toward the traditional bare metal stents and the newly developed drug-eluting stents, where the goal of the coating is to prevent secondary blood clotting. In the latter case, we address the ability to control the release of macromolecules that were attached to the surface of the polymers that were intended for use in topical applications, such as wound dressing. Another function of surface grafting of macromolecules includes tissue engineering for the promotion of cellular responses. In addition to the surface grafting of macromolecules, we review the types of macromolecule-grafted surfaces and their interfacial effects with surrounding tissues. In general, our review provides an update on current technologies and trends in thrombosis prevention and dressing for wound healing. By using polymers with modified surfaces, which were serving as active sites for biomedical functions, the device can achieve their therapeutic performance for the disease states.

## 2. Surface Functionalization of Biomaterials

Surface functionalization of polymers by grafting (“graft polymerization”) is of utmost importance in biomedical, environmental, and industrial applications [4,5,6]. When new functionalities are introduced task-specifically onto polymer surfaces, the polymer’s physicochemical, morphology, and biocompatibility properties are changed [6,7,8]. Graft polymerization of monomers onto substrate surfaces can be achieved by both chemical and physical procedures. Over the past two decades, various techniques have been developed to perform graft polymerization onto different surfaces [9]. The most commonly used graft polymerization techniques for biomedical applications are described in this section. 

### 2.1. Grafting by Chemical Means

Grafting by chemical means can be distinguished into three major pathways: free radical, ionic, and living polymerization [10,11,12]. In chemical grafting, the initiator, which is a chemical, is significant, since it affects the path where the grafting process occurs [11,13,14]. A polymer can be grafted under various conditions of the monomer, including an aqueous solution, gas phase, or plasma phase of the monomer [11,14,15]. The major limitation of chemical grafting is the formation of homopolymers as byproducts, which are difficult to separate from the grafted polymers [16,17,18]. On the other hand, the advantage of grafting by chemical means is that it modifies the surface properties and not bulk properties [19]. During the free radical grafting process, radicals are created from the initiator chemicals and repositioned to the substrate polymer, which then reacts with the monomer to form graft polymers. Free radicals can be either generated directly on the initiator chemical or indirectly via the redox reaction [20]. In the ionic grafting process by chemical means, initiator chemicals produce cationic or anionic centers that initiate graft polymerization [21]. The third grafting technique by chemical means is living polymerization, which is also referred to as a controlled process. During this polymerization process, chain growth is accomplished by an active propagation group that has no or negligible effect on chain transfer or termination reactions [16,22]. Therefore, this polymerization is more controlled than conventional radical polymerization as it also produces more uniform grafted polymers with regulated molecular weights [16,23,24]. Furthermore, during the whole living polymerization process, the concentration of the active propagation species remains consistent [11,16,22]. Likewise, there is a linear correlation between the amount of monomer that is converted and the molecular weight of the grafted polymer [11,16,22,24].

### 2.2. Plasma-Induced Grafting

Plasma treatment is an efficient way to add functional groups to biomaterial surfaces, create closer pore membranes, and alter nanostructure membranes symmetrically [25]. In this method, the substrate surface is subjected to high energy radiation by electron-induced excitation, ionization, and dissociation processes [26,27]. The excited plasma electrons stimulate chemical bond cleavage in the polymeric structure. This, in turn, leads to the formation of macromolecule radicals, which then initiate graft polymerization. Plasma treatment can improve the hydrophilicity by increasing the number of polar groups on the biomaterial surface [28]. Another advantage of this technique is the modification of the chemical composition without altering the material properties of therapeutic devices. Upon the introduction of reactive sites on the medical device, numerous methods such as self-assembly, non-covalent physisorption, and covalent binding of polymer brushes can be used to attach the desired polymeric coating. 

### 2.3. Ultraviolet-Induced Photo-Initiated Grafting

Ultraviolet radiation is an inexpensive, clean, and straightforward method for surface grafting of polymers [29,30]. This technique eliminates the need for initiators and washing steps [31,32]. During ultraviolet (UV) irradiation, free radicals are generated by use of UV light in only a few steps to produce a graft product [29,31]. Briefly, the parent polymeric surface is activated by UV radiation producing free radicals and allowing attachment of monomers. Thus, creating a functionalized polymer surface that has specific characteristics, such as increased adhesion properties and surface wettability [29,31,33]. Ultraviolet functionalization of polymer surfaces can be mainly attained by two methods. In the first method, the chemical reaction takes place upon irradiation of the polymer in the direct presence of a monomer solution. The second method is based upon pre-irradiation. During this technique, a monomer solution is exposed to a pre-irradiated parent polymer that has reactive radical sites or peroxides on the surface. These active species on the substrate polymer have the potential to induce a chemical reaction with the monomer solution [29,30]. The limitation of photo-initiated grafting is that the irradiation is non-penetrative, which makes it only suitable for surface modification [31,34].

### 2.4. Radiation Grafting

Polymeric structures and other macromolecules can also be changed by high energy γ-radiation, which causes the induction of radicals, ions, and free electrons. These created reactive sites on a polymer backbone can react with a monomer producing a grafted polymer [16,35,36]. In comparison with chemical grafting, radiation-induced grafting is a clean method since it does not require initiator chemicals, which can cause contamination. Additionally, washing steps are not required. Furthermore, in contrast with photoinitiated grafting, it penetrates deeper [16,35,36,37]. The disadvantage of radiation grafting is that severe degradation and decomposition of the polymer can occur [38,39,40]. There are two types of radiation grafting, namely free-radical grafting and ionic grafting. 

In the first method, the irradiation of macromolecules can cause homolytic fission; thus, resulting in the formation of free radicals on the polymer [36]. Although an initiator is not essential in this process, the medium is. In case the irradiation is performed in air, there is a possibility that peroxides are formed on the polymer because of oxygen in the atmosphere [35]. The backbone nature of the polymer determines the lifetime of the free radical. Free-radical grafting can be performed in three different ways: (a) pre-irradiation, (b) peroxidation, and (c) the mutual irradiation technique. During the pre-irradiation procedure, parent polymer is first irradiated in a vacuum or in an inert gaseous environment to create free radicals [36,38,41,42]. Next, to initiate grafting, the irradiated polymer is combined with the monomer in liquid or gas state, or as a solution present in a solvent [36,38,41]. In the peroxidation procedure, the polymer backbone undergoes high energy radiation in the presence of air or oxygen to develop stable hydroperoxides or diperoxides. Grafting is then initiated when these peroxy products are reacted with the monomer at high temperatures, which in its turn leads to the decomposition of the peroxides to form radicals [38,39]. In the last procedure, mutual irradiation, which is the parent polymer, and the monomer are irradiated concurrently to produce free radicals and successive grafting [41].

Finally, ionic grafting is based upon the irradiation of the polymer to generate the polymeric ion (cationic or anionic), and subsequently, it is reacted with the monomer to produce the grafted polymer [34,36]. Ionic grafting is known to have a high reaction rate. Consequently, small radiation doses or times can form a considerable amount of grafting [36,38,41]. 

### 2.5. Enzymatic Grafting

In this grafting method, radicals are generated by enzymes. Enzymatic grafting is environmentally clean and has a high specificity; thus, allows for the synthesis of almost pure modified products without homopolymers [7,43,44,45]. One category of enzymes often used in the grafting of biopolymers is the oxidative enzymes (e.g., laccases, peroxidases). These enzymes can produce radicals on parent polymers, which can subsequently react with the monomer to complete the grafting process in a domino type reaction [46,47]. Some of the shortcomings of enzymatic grafting are that for optimal enzyme activity, enzymes need narrow pH, temperature, and concentration ranges. Moreover, for a chemical reaction to take place, the microenvironment needs be in a liquid state at the optimal pH and temperature for the specific enzyme being used [37,44,45,48].

### 2.6. Nanoparticle Grafting

The use of nanoparticles to modify the polymer surface is an effective way to enable cell adhesion [49]. There is a wide variety of materials, each with its advantages and disadvantages, which can be employed to form nanoparticles. The noble metals such as silver and gold have unique properties and are among some of the most commonly used materials for the fabrication of nanoparticles [50,51,52]. Numerous variables of the nanoparticles need to be taken into consideration, such as shape, size, and dispersion when nanostructure surfaces are created for a variety of biomedical applications. The nanoparticle dispersion is one of the most crucial and challenging factors to control because nanoparticles tend to agglomerate due to their extremely high surface energy. Therefore, choosing a suitable medium for the formation of a nanoparticle solution or an additional treatment of the surface is usually needed [53]. Altogether, to ensure appropriate interactions and sufficient bonding sites for the grafted nanoparticles, it is first necessary to modify the morphology and the physicochemical properties of the surface [54].

### 2.7. Laser Treatment

Laser-induced periodic surface structures (LIPSS) can be formed under specific conditions when solid substrates are treated with laser irradiation [55]. These LIPPS have various shapes and forms, and they can be subdivided into low spatial frequency and high spatial frequency. The low spatial frequency LIPSS have a period similar to the wavelength of the laser beam and are usually formed on macromolecular substrates [56]. For the formation of these LIPPS on polymer substrates, such as foils, a good absorption in the wavelength region of the incident laser beam is required [57]. Therefore, LIPPS have often been detected on macromolecules composed of strong absorbing functional groups and conjugated systems. The most common types of LIPPS that can be formed on polymers are ripples. In general, it is assumed that the ripple formation takes place when an incident beam interferes with a perpendicular reflected beam. This interference leads to an accumulation of energy, which results in a temperature difference between the non-crystalline and the crystalline phase of the polymer substrate, causing the material to flow from high to lower temperature areas. Consequently, generating a periodic pattern on the macromolecular surface [56,58]. The influence range of the periodic pattern formation and the dimensions of the ripples generally depend on the type of polymer and the conditions of the laser treatment [56,59]. Laser treatment changes the surface energy of the polymer substrate, which results in increased cell adhesion and proliferation [60]. Moreover, when the laser fluence is extremely below the ablation threshold, the surface chemistry changes causing an increased cell adhesion. Laser radiation can also break chemical bonds, resulting in the formation of highly reactive radicals on the polymer surface, which rapidly react with the nearby atmosphere, leading to surface oxygenation and the formation of new functional groups [61]. Lastly, the second LIPPS group, the high spatial frequency, have a period that is much smaller than the wavelength of the incident laser beam and are more seen on laser-treated semiconductors and metals [56].

### 2.8. Ion Implantation

The biocompatibility of a polymer substrate can also be modified using ion implantation. This method has the capability of increasing both the adhesion and the resulting proliferation of cells [62]. It is based on the implantation of high energy ions into the substrate surface. Prior to implantation, these ions are separated using a magnetic field and are then accelerated in an electric field. Afterward, the energy of the ions rapidly disperses and breaks down the polymer chains leading to an increased concentration of highly reactive radicals in the substrate surface. Altogether, these radicals cause an increased reactivity of the surface layer, as well as to crosslinking of the fragmented polymer chains, double bond formation, and the evolution of gaseous degradation products [63,64]. The type of ions employed in biomedical applications is from noble gases and common elements (e.g., nitrogen, oxygen) that are found in biological tissues [65,66]. The ion implantation penetration debt is dependent on the weight and energy of the ions [67].

## 3. Antifouling Biomaterials

Contaminations from various proteins and microbicides on the surface of implantable and injectable devices, which are known as biofouling, were responsible for 26% of device failure in 2011, according to the Centers for Disease Control and Prevention in the United States (U.S.) [68]. To prevent biofouling-related morbidity and mortality, the inhibition of protein deposition on the surface of these devices appears to be mainstream in antifouling biomaterials [68]. Specifically, biofouling is a process in which the surface of biomaterials is covered with organisms and their byproducts that limit the performance of biomedical devices [69]. The successful development of biomedical devices is based on innovations in surface sciences and technologies in creating nano-scaled surface textures to control the interactions between the biomedical devices and the surrounding biological milieu. As a result, current bioengineering research has focused on the attachment of macromolecules on the surface of biomedical devices to provide a fouling resistant surface that inhibits specific biological responses (e.g., platelet deposition in vascular stents and biofilms on wound dressing materials) [70]. Surface decoration of macromolecules on biomedical devices may provide the correct chemical and the physical properties for the biomaterials to prevent the occurrence of biofouling [69].

### 3.1. Poly(ethylene glycol)

Poly(ethylene glycol) (PEG) is a fouling-resistant material that functions by reducing the binding affinity of protein molecules to the surface of membranes [71,72]. For example, the surface poly(ether sulfone) (PES) membrane was grafted by poly(ethylene glycol) methyl ether methacrylate (PEG) with vinyl amides, including *N*-vinylformamide (NVF), *N*-vinylacetamide (NVA), or *N*-methyl-*N*-vinylacetamide (MVA) for comparison in antifouling behaviors [73]. Results showed that PEG-grafted surfaces at a concentration of 0.08 mol/L exhibited a lower fouling index (R = 0.3 ± 0.03) than the control PES membranes (R = 1). In addition, the fouling index further decreased after surface grafting of PES membranes with binary mixture of PEG and vinyl amides, where PEG-NVA showed the lowest fouling index (R = −0.56 ± 0.29) at PEG and NVA concentrations of 0.06 mol/L and 0.14 mol/L, respectively. These results demonstrated that binary systems promoted antifouling properties in PEG, which makes it applicable for medical and marine uses. Other fabricated copolymers of poly(vinyl chloride-*co*-poly(ethylene glycol)methyl ether methacrylate) with varying poly(ethylene glycol)methyl ether methacrylate (PEGMA) segment percentages for applications in antifouling microfiltration membranes [74]. Increasing PEGMA segment percentage improved membrane hydrophilicity and antifouling property due to a high level of PEGMA concentration on the surface of the membrane. Bovine serum albumin (BSA) adsorption on the membranes with varying PEGMA segment percentages suggested a satisfactory antifouling effect of 9.8 mol % of PEGMA in the membrane. Later, the authors fabricated membranes from blends of poly(vinyl chloride-*co*-poly(ethylene glycol)methyl ether methacrylate) and poly(vinyl chloride) [75]. Results suggested a significant improvement on membrane hydrophilicity and antifouling properties when the blend ratio of poly(vinyl chloride-*co*-poly(ethylene glycol)methyl ether methacrylate) and poly(vinyl chloride) was greater than 1:2. In general, PEG shows excellent properties in antifouling, which makes PEG an ideal candidate for coating materials on biomedical devices.

### 3.2. Hyperbranched Fluoropolymers

Fluoropolymers have been extensively researched on antifouling properties due to their low surface energy, low wettability, and chemical stability [76,77,78]. Studies showed that coatings that are made from hyperbranched fluoropolymers cross-linked with bis-amino-propyl PEG and bis-amino-propyl polydimethylsiloxane (PDMS) exhibited excellent antifouling properties [79]. In particular, (BSA) adsorption tests suggested a 60% greater resistance to protein adsorption in comparison to the fouling performance of a commercially available anti-biofouling silicone coating. Other fabricated coating materials from cross-linked hyperbranched fluoropolymer and PEG with PEG weight percentages ranged from 14% to 55% to form an amphiphilic network [80]. The cross-linked hyperbranched fluoropolymer PEG coatings showed to be resistant to BSA adsorption, a lectin, and lipopolysaccharides from *E. coli* and *Salmonella minnesota* when comparing to the control surfaces. Interestingly, even though cross-linked hyperbranched fluoropolymer and PEG at 55 wt % received the highest hydrophilicity, the 45 wt % PEG coating was the most effective in resistance to protein and lipopolysaccharide adsorption, suggesting a possible compositional range for the cross-linked hyperbranched fluoropolymer PEG coatings. Overall, the excellent surface properties and the chemical heterogeneity in hyperbranched fluoropolymers allow for them to serve as a class of hardy and complex surfaces that exhibit characteristics of antifouling behaviors.

### 3.3. Zwitterions

Grafting zwitterions on membrane surfaces is another method that is used to improve antifouling properties in biomedical devices [81,82,83,84,85]. In a study, polyimide membranes that were grafted with zwitterions where the results showed improvements in membrane hydrophilicity, fouling resistance, and permeation flux [86]. In addition, the BSA fouling test showed an increase in flux recovery ratio and reversible flux decline ratio, suggesting that polyimide-zwitterion membranes performed better with antifouling properties as compared to the blank polyimide membranes. In another study, a thin layer of coating with bacterial resistant properties was fabricated from tannic acid and zwitterionic polymer [87]. The coating showed antifouling behaviors in resistance of protein absorption, platelets, and bacteria adhesion. Others used natural sulfur-containing compounds, such as l-cysteine (Cys), l-methionine, and glutathione (GSH), for the fabrication of surface zwitterionic ligands to prevent protein nonspecific adsorption on gold substrates [88]. Results suggested that coatings that were made from Cys and GSH displayed a high hydrophilicity, whereas coating made from GSH showed the best performance in resistance to BSA adsorption. However, the drawbacks with zwitterionic materials include difficulty in mass production, non-biodegradable properties due to strong carbon-carbon backbones, and cytotoxicity/biocompatibility issues in drug delivery systems [89].

## 4. Nanostructured Film Surfaces 

### 4.1. Canonical Thrombogenic Cascade

It is well-established that blood coagulation follows a canonical enzymatic “cascade” of events [90]. This extracellular process typically begins when damage to the vasculature results in the exposure of flowing blood to underlying adventitial cells, which constitutively express an extracellular membrane-bound protein called Tissue Factor (TF). TF acts as an essential and potent cofactor for the “first” enzyme in the cascade, Factor VIIa (FVIIa). As TF is an integral membrane protein, the enzymatic activity of the resulting FVIIa-TF complex is expressed in two dimensions on the cell surface [91]. Localization of the coagulation reactions to the site of damage is enhanced by the dependency of several subsequent enzymatic reactions on phospholipid surfaces that are provided by damaged cells or activated platelets. The entire coagulation system has thus been optimized for surfaces. This poses a problem when artificial surfaces are introduced into the systemic circulation, as an alternative mode of triggering blood coagulation can also occur through what has been termed “contact activation”, which occurs independently from the presence of TF, but it progresses through much the same enzymatic pathways and results in the same product—a clot or fibrin deposition. Since many of the byproducts of coagulation reactions affect the inflammatory response, which is an additional major determinant in the rejection of implants or artificial devices, controlling or limiting these procoagulant enzymatic responses (regardless of their ability to form a clot) would address both issues. Thus, the generation of surfaces that are anticoagulant in nature or that resist (either passively or actively) these enzymatic procoagulant reactions is of great interest.

The down-regulation of active coagulation enzymes and their complexes typically follows the natural off-rates of these proteins from their surfaces. Released enzymes are typically rapidly inhibited and/or captured in the natural flow of the circulation and are carried away downstream. This effectively dilutes them to levels that are ineffective long enough to allow for them to be filtered and removed from the circulation. In theory, this natural removal process can potentially be leveraged by designing hemocompatible surfaces that also contain tethered reversible inhibitors. While the hemocompatibility of the surface could use existing technology, the addition of the reversible inhibitors to these surfaces would generate actively anticoagulant surfaces that can be designed to respond to the rise and fall of procoagulant proteins that are based entirely on their kinetics of association and dissociation. This reversibility would allow for the slow release of captured enzymes only as the local levels are reduced to sub-coagulant levels through natural removal mechanisms. This slow release would not be to allow for coagulation, but rather to mediate the slow release of the captured enzymes back into the circulation to be removed by natural means; effectively making a renewable inhibitor surface. This differs drastically from current inhibitor surfaces that have largely, if not entirely, relied on the irreversible inhibition of enzymes, and thus display reduced efficacy as the inhibitor is utilized and not regenerated.

Other mechanisms could also be envisioned where releasable engineered protein inhibitors/receptors could be used to target specific factors irreversibly. However, the accumulated complexes could be “released” from the surface through the injection of a specific release reagent, and then the surface repopulated with fresh inhibitor/receptor in a subsequent injection. Although these surface regeneration models would require safe, highly specific, high-affinity interactions, the technology is currently available and it should be considered as a viable mode of engineering regenerative surfaces.

### 4.2. Films for Thrombosis Applications 

Vascular stents are an implantable biomedical device that interacts with blood and tissues constantly. They have extended the life of many patients who suffer cardiovascular diseases. However, studies have shown that metal-based stents are likely to promote secondary blood coagulation as time progresses, which would require an additional surgical procedure to remove the old stent and replace with a new stent [92]. To address the issue of thrombosis on metal-based stents, anticoagulant films/coatings become a viable strategy to extend the use of and increase the thromboresistant behavior of stents [93]. Surface modifications of coatings/films through the use of nanotechnology further create new possibilities to achieve outstanding properties, both physical and chemical, to resist blood clotting.

#### 4.2.1. Nano-Structured and Roughened Surfaces

In an effort to find new methods in order to solve the coagulation problems with the metal-based stents, polymers are being used to coat the surface of the stents. However, certain polymers showed non-adhesive behaviors to the metals being used. Altering the surface profiles of the stent material by intentionally creating nano-scaled roughness can improve the adherence of the polymeric coatings and its blood compatibility (Table 1). In a study, nickel-titanium (NiTi) substrates were nano-roughened using target-ion induced plasma sputtering (TIPS) technique, where the nano-roughened surface was then coated with a polytetrafluoroethylene (PTFE) layer by spin coating with 60 wt % of PTFE [94]. The surface structure of NiTi created by TIPS process provided a greater number of infiltration sites for the PTFE, allowing for better bonding of the PTFE to the NiTi substrates. Another study suggested the use of four-beam laser interference method to alter the surface profile of the metal stent [95]. The modified metal surface was then coated with a functional polymer layer of phosphorylcholine that received a tethered biomimetic layer of miRNA126. The micro/nano-structured bionic stent was then loaded with a drug to achieve a sustained release behavior for coronary heart disease.

The use of nanotextured materials has attractive applications in the biomedical field that range from medical implants to biosensors. In a study, the surface of Ti6A14V alloy is altered by using a thin nano-structured film of zirconium oxide in a plasma ion implanter [96]. Results showed that the deposition of zirconium oxide improves the wear resistance of the Ti6A14V alloy, allowing for the use in medical implantation. Another study demonstrated the modification of the surfaces of titanium implants to promote the biological responses of mesenchymal stem cell (MSC) to enhance osseointegration [97]. The modified surfaces of titanium implants include nano-scale TiO_2_ topography (TNT), micro-scale sand blasted-acid etched topography (SLA), and hybrid sand blasted-acid etched/nanotube topography (SLA/TNT). Results suggested that MSCs that were cultured on TNT had cell elongation of approximately 3.8 with enhanced osteogenesis, making it favorable for in vivo applications. TNT also showed promotion of MSC adhesion, proliferation, and differentiation. Others used stainless steel decorated with high ordered self-organizing nanopores with gold nanostructures (AU/NPSS) for electrochemical biosensors to determine levels of dopamine [98].

#### 4.2.2. Macromolecule-Grafted Surfaces

Radiation grafting is a popular method to attach macromolecules to a film surface, where the chemical and physical properties of the surfaces can be altered, allowing for a wide range of biomedical applications. In a study, methacrylic acid (MAA) was grafted by either argon plasma or UV light to thermoplastic polyurethane (TPU) to compare their blood compatibility and cell adhesion with human bone marrow cells (HBMC) [99]. Results suggested that both plasma and UV grafted MMA surfaces showed higher water contact angles than the unmodified TPU surface for 30 days, suggesting the stability in hydrophobicity. However, the hemolysis index (HI) suggested that UV grafted MMA surfaces were non-hemolytic (0 > HI > 2) as compared to the plasma grafted counterparts (HI > 5). In addition, UV grafted films exhibited a slightly larger O/C ratio than the plasma grafted films. Due to the excessive carboxylic (COOH) groups at the surface of the film, cell adhesion reduced on UV grafted film than the plasma-grafted films. In a similar study, 2-hydroxyethyl methacrylate (HEMA) was grafted by the similar plasma and UV processes using the same TPU surfaces for applications in heart valves [100]. The grafting of HEMA onto TPU surfaces using plasma treatment suggested a better biocompatibility with low thrombogenicity.

**Figure 2:**
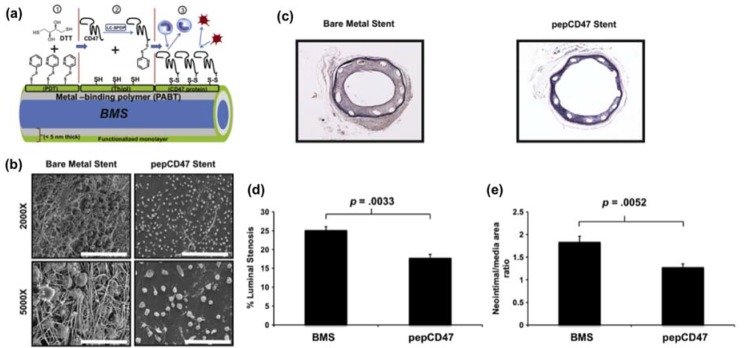
(**a**) Schematics of chemical modifications to attach CD47 proteins on the surface of bare metal stents. (**b**) Comparison fibrin deposition and trapped cells using scanning electron microscope (SEM) images on surface characterizations of an explanted endovascular stent in a rat carotid artery after 30 min. Scale bar = 30 μm. (**c**) Histological sections of CD47 grafted stents and bare metal stents demonstrating anti-restenotic effects in a rat carotid artery after 14 days. (**d**) Quantitative morphometric analyses on a Hematoxylin-eosin stain of explanted stents showing luminal stenosis. (**e**) Quantitative morphometric analyses on a Hematoxylin-eosin stain of explanted stents showing Neointima/media area ratio due to CD47 immobilization. Reproduced with permission from [101].

A more novel approach to surface modification is through the attachment of bioactive components (proteins and enzymes) to the surface of a film (surface decoration). One study showed that modifying the surface of a stent by immobilizing CD47 proteins can reduce inflammation in the body during stent deployment (Figure 2) [101]. To create these surfaces, polyallylamine bisphosphonate-modified 316L-grade stainless steel surfaces were thiolated with CD47 at the surface. The modified surfaces showed the significant reduction of cell attachment than the unmodified, scrambled control peptide, and BSA-modified surface, indicating an inhibition of inflammatory cell attachment to the surfaces of the steel. In vivo efficacy study suggested an inhibition of cellular inflammatory response when using CD47 grafted surfaces. The modified surface can be characterized as a bioactive surface suitable for specific needs, with the ability to reduce inflammatory responses and restenosis.

Graft stenosis and occlusion are a result of neointimal hyperplasia that is caused by smooth muscle cells (SMCs). Even though SMCs are necessary for the structure and the function of the neovessel, its abnormal proliferation can damage the vascular graft [102,103]. Transforming growth factor-beta (TGF-β) and platelet-derived growth factor (PDGF) have shown their ability to suppress the pathway of SMCs in neointimal hyperplasia [103]. A research demonstrated that seeding tropoelastin onto the luminal surface of the vascular craft inhibit neointimal hyperplasia in a mouse model [103]. Results suggested that tropoelastin seeded vascular grafts had remarkably less SMCs growth in the surrounded tissues as compared to the blank vascular grafts after eight weeks of implantation.

#### 4.2.3. Composites and Inorganic Coatings

**Figure 3:**
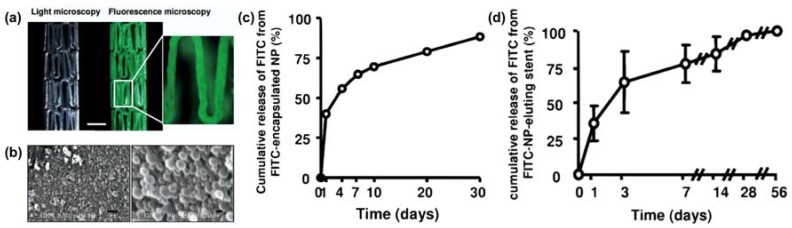
Fabrication of a fluorescence marker enabled nanoparticle-eluting stents using cationic electrodeposition coating technology. (**a**) Light and fluorescence microscopic images of fluorescence marker enabled nanoparticle-eluting stents. Scale bar = 1 mm. (**b**) Scanning electron microscopic image showing the nanoparticle-eluting stents (scale bar: left = 1 μm; right = 100 nm). (**c**) In vitro cumulative release profile of Fluorescein Isothiocyanate (FITC) (a fluorescence marker) encapsulated nanoparticles. (**d**) In vitro cumulative release profile of FITC (a fluorescence marker) from nanoparticles-eluting stents. Reproduced with permission from [105].

Drug-eluting stents, which are a popular device that loads drug on the surface of the stents, are one the first generation of stents with nanostructures made to combat blood coagulation. One study aimed to create a biodegradable multilayered polymeric surface coating using poly(d,l-lactide-*co*-glycolide) and polycaprolactone with nanopores for drug-eluting stents to increase biodegradability [104]. Results on the release profiles suggested four stages of the release characteristics, including, a burst release (5 h), an exponential release (1 week), a sustained release (2–7 weeks), and an increased release (7–10 weeks). Another study focused on creating a system that utilizes nanoparticle-eluting stents for drug delivery (Figure 3) [105]. The stent coating was created using cation electrodeposition with poly(d,l-lactide-*co*-glycolide) nanoparticles and fluorescein-isothiocyanate. The nanoparticle-eluting stents showed no effects that were related to stent-induced injury, inflammation, or endothelial regeneration, concluding that the device had no vascular toxicity and was compatible with human subjects.

A number of problems can arise from the methods and materials to produce coatings on vascular stents, including chemical synthesis, biological treatments, or the use of polymers materials. It has been noted that late stent thrombosis, wear of the implant, inflammation, and other complications can be caused by these variables. One approach to these issues is to create a superhydrophobic surface, which has excellent blood compatibility. In a study, surface hydrophobicity was improved by the dip coating of carbon aligned nanotube films with polycarbonate-urethanes (PCU) of different fluorinated alkyl side chains of 20% and 50% [106]. The wettability of the PCUs increased from 109° for blank PCU (20%) to 164° for nanotubes coated PCU (20%) with a similar trend being observed for the PCU groups having 50% of fluorinated alkyl side chains. In platelet adhesion, the results suggested that nanotube that was covered PCU films had outstanding platelet anti-adhesion properties. It was also found that if naturally activated platelets in the body were present more than 75%, those platelets were reduced with the introduction of the nanostructured polymer. These findings showed that nanotubes are good candidates for coating materials on implantable stents.

In an effort to reduce blood clot formation from the use of polymers materials, several studies have taken a new approach of non-polymer-based coatings to reduce the risks of inflammation. One study used magnetic mesoporous silica nanoparticles (MMSNs) and carbon nanotubes (CNTs) to coat the stents [107]. Results on mechanical properties showed less flexibility as compared to the polymer counterparts due to the inorganic nature of the materials. Hemolysis assays showed less than 5% of hemolysis percentage, while platelet-adhesion tests revealed a lower potential for thrombosis than the 316L-bare metal stents (BMS), suggesting good blood compatibility. In vitro drug release study displayed a sustained release profile for rapamycin (RAPA). When comparing the MMSN- and CNT-coated stents with the commercial polymer-coated RAPA-eluting Firebird-II stent, a significantly better re-endothelialization was found on the MMSN- and CNT-coated stents in the early stages than the Firebird-II stents. Others have created a non-polymer coating with nanopillar array textured surfaces from medical grade MP35N alloy wires (35% Co-35% Ni-20% Cr-10% Mo in wt %) [108]. The non-polymer coating stents indicated superior endothelial cell growth, more continuous monolayer formation, and an overall improved endothelialization, demonstrating a novel concept that reduces late stent thrombosis.

### 4.3. Films for Drug Delivery Applications

Films are a fundamental component in drug delivery systems and they are widely used in pharmaceutical industry. The design of an ideal drug incorporated film will be responsible for the temporal and spatial control-release of drugs without harmful effects on tissues. Therefore, the functions of films in drug delivery systems are to target the site, shelter a drug for a long enough period of time, and release it in a controlled manner [109]. There are some critical factors for the consideration of film fabrication used in drug delivery, including the physicochemical properties of the polymers and drugs, anatomical and physiological limitations, and methods and formulations. Materials that are biocompatible and biodegradable have been formulated for drug-incorporated films with the purpose to efficiently deliver drugs to a human tissue [109,110,111].

#### 4.3.1. Oral Drug Delivery

Natural and synthetic polymers have been used as colon delivery drug options with the ability to withstand the degradation by the local bacteria species in the stomach and small intestine digestion. Several carbohydrate polymers, such as pectin, chitosan, guar gum, dextran, and alginate have been developed into drug delivery systems for colonic applications, with the possible use of a crosslinking agent to enhance their efficiency in drug delivery system [112]. In another study, various polymeric films consisting of a starch derivative Nutriose were used to coat the pellet for drug delivery on Crohn’s disease and ulcerative colitis patients [113]. In vitro release of 5-aminosalicylic acid suggested a positive correlation with the thickness of the coating. In addition, increasing the blend ratio of ethylcellulose to Nutriose increased the ability of a sustained release of the drug. Others used pectin/ethyl cellulose film to coat pellets containing 5-fluorouracil for sustained release purpose [114]. In vitro release showed that the coated pellet achieved 34% release of 5-fluorouracil in 24 h, whereas the addition of rat cecal contents increased the release to 85%. In plasma, the coated pellets had eight times less of mean drug concentration than the uncoated controls, whereas the coated pellets had 10 times higher of mean drug residency than the uncoated controls. Overall, polymeric films have demonstrated the ability to sustain the release of drug in oral dosage. 

#### 4.3.2. Grafted Surfaces for Topical Applications 

Advances in biotechnology have enabled techniques and methods to modify the surface of films for drug delivery in topical applications. This process is achieved by graft polymerization to fabricate materials with complex polymeric structures with desirable physicochemical and biological properties, such as biodegradable, biocompatible, and antibacterial properties [115,116]. 

Stimuli-responsive polymers are attractive in drug delivery since they respond sharply to physical or chemical condition changes, allowing for a programmable release of a drug (Table 2). Such stimulations include environmental changes in a physical (e.g., humidity, temperature, high pressure, ionic strength, solvents, high pressure, radiation, light, electric, and magnetic fields) and/or chemical (e.g., pH, specific ions, enzyme substrates, and biochemicals) manner [116,117]. Poly(acrylic acid), poly(methacrylic acid), and poly(*N*-isopropylacrylamide) have been widely used as a stimuli-responsive polymer for nasal, ocular, oral, and vaginal drug delivery.

### 4.4. Nanotextured Films in Other Biomedical Applications

Polymeric films are widely used for substrate materials in cell cultures, where the surface properties of the films play important roles in determining cell growth and behaviors. For example, chitosan films were used in studies to culture rabbit corneal keratocytes [134,135]. Results suggested that surface properties as well as bulk properties, as mediated by the degree of deacetylation in chitosan, significantly affected the adhesion, spreading, migration, proliferation, and differentiation of rabbit corneal keratocytes. In particular, cell adhesion and spreading increased with increasing crystallinity in chitosan films. The increase in crystallinity decreased the surface roughness, while increasing the surface stiffness of the film, resulting in a decrease of cell migration and an increase in cell proliferation. More importantly, the level of gene markers increased significantly during mass production of the cells via the proliferation stage, suggesting that the cells maintained their phenotype without differentiating into fibroblasts and/or myofibroblasts. In addition, others also observed the similar correlations between surface roughness and cell migration and proliferation [136,137,138,139], which was supported by increased adsorption of fibronectin, which is a pre-requisite for cell attachment and growth [140]. In general, the surface texture of a substrate can significantly determine cellular behaviors where the cultured cells may be used for cell therapy in ocular diseases. 

In bone tissue engineering, the complex reactions occurred at the interface of the biomedical implants and the surrounding tissues determine the osseointegration and the long-term success of the implant [141]. A study demonstrated that osteoblast cell adhesion capacity to a glass and titanium surface was promoted by an adhesive peptide (HVP) grafts onto the surface of the materials [142]. Results suggested a significant increase of osteoblast adhesion when compared to a non-adhesive peptide and silanized glass after 2 h of culture. In addition, surfaces that were grafted with a retro-inverted dimer peptide (D-2HVP) also showed an increase of cell adhesion as compared to the controls. Others covalently grafted endothelial and osteoblastic cells, which were cultured on hybrid materials, with different densities of RGD (Arg-Gly-Asp) assemblages of tripeptides onto the polymer surface [143]. The cell adherence property of RGD was affected by the presence of integrin receptors on this tripeptide combination. Results from cultures of newborn mouse osteoblast cells (MC3T3-E1) and human saphenous veins endothelial cells (HSVEC) after 6 h suggested a strong focal adhesion with grafted peptide surfaces, where increasing peptide grafting density increased the level of focal adhesion from the cultured cells.

## 5. Nanostructured Fiber Surfaces

Fibers that were electrospun from polymeric solutions have received great interest in the recent years. Owing to the high surface area to volume ratio, electrospun fibers exhibit potential applications in tissue engineering scaffolds [144], drug delivery carriers [145], and biomedical devices [146]. In addition, fiber surfaces can be decorated with functional macromolecules to improve characteristics in cell signaling. In this section, we review the surface functionalization of electrospun fibers for wound healing applications, and the use of surface texture from electrospun fibers for tissue engineering applications.

### 5.1. Surface Nanostructure for Wound Healing

**Figure 4:**
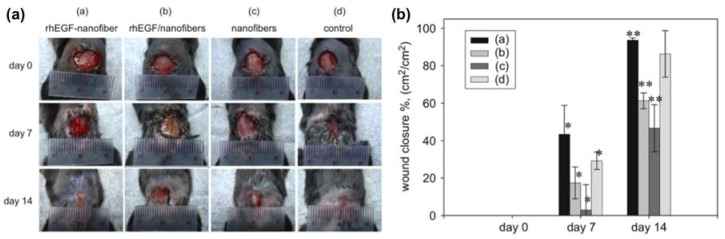
(**a**) In vivo evaluation of wound healing using a mouse model for comparison of epidermal growth factor-immobilized nanofibers with blank nanofibers and controls. (**b**) Quantitative analysis of wound-closure rates in the diabetic mice for epidermal growth factor-immobilized nanofibers with blank nanofibers and controls. * and ** indicate statistical significances *p* < 0.05. Reproduced with permission from [147].

Proteins, such as growth factors, are essential to the wound healing process due to the ability in promoting cell cycles that accelerate wound healing. The delivery of these large macromolecules via electrospun fibers is of particular interest since fibers possess several advantages when compared to current dressing materials, especially with the ability to control the release of macromolecules to promote wound healing (Figure 4) [147]. Typically, these macromolecules are immobilized on the surface of the fibers, where their release characteristics can be activated through diffusion (breaking of the immobilization bonds) and/or through the dissolution or degradation of the fibers [148]. For example, the controlled releases of basic fibroblast growth factor (bFGF) and epidermal growth factor (EGF) were demonstrated from polycaprolactone (PCL) and polyethylene glycol (PEG) coaxial fibers [149]. Results suggested that only 2% of the immobilized EGF was released in a week from the PCL-PEG shell, whereas 30% of bFGF was released in 12 h when encapsulated in the core. Others showed the ability to control wound infections via the antibiotic efficacy of human cathelicidin peptide LL37, where Cysk-KR12, an antimicrobial peptide motif (Cys-KR12) originated from LL37, immobilized fibers were able to maintain antibacterial properties for three weeks [150]. The study inferred the important role of Cysk-KR12 in wound healing by activating keratinocytes, fibroblasts, and monocytes.

The immobilization of proteins and peptides on the surface of electrospun fibers promotes cell growth in tissue engineering. In a study, peptide sequence E7 was immobilized on electrospun PCL fibers to promote the formation of mesenchymal stem cells (MSCs) [151]. The in vivo rat model showed that MSC growth on the PCL/E7 fibers achieved a higher percentage than the Arg-Gly-Asp peptide (RGD) control group with less inflammatory cells. The other immobilized soluble eggshell membrane protein (SEP), which was derived from natural eggshell membrane, on the PCL fibers for tissue engineering scaffold applications [152]. Results suggested that SEP-grafted PCL fibers were more hydrophilic than the blank PCL fibers, which further promoted the attachment, spreading, and proliferation of human dermal fibroblasts (HDFs) as compared to the untreated material.

**Figure 5:**
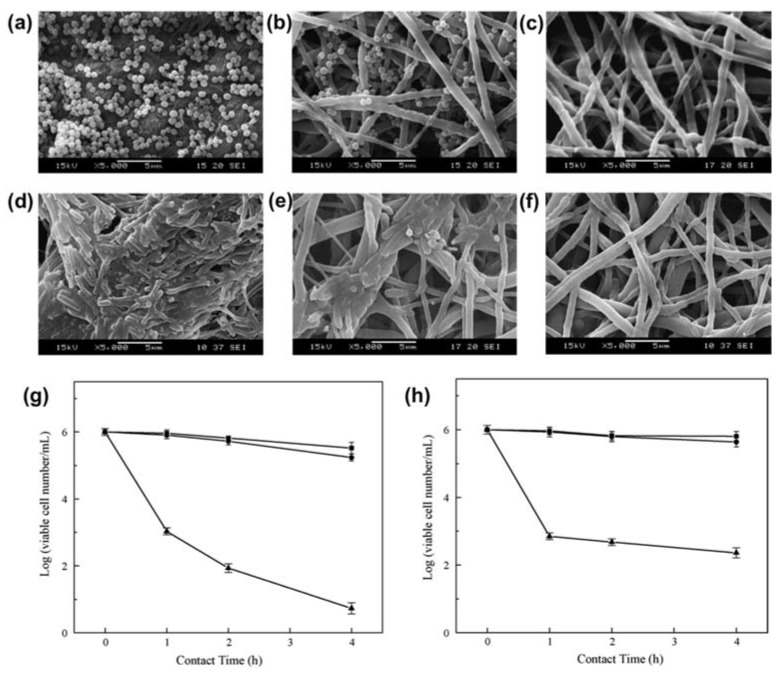
Scanning electron microscopic images of (**a**,**d**) filter paper (control), (**b**,**e**) pristine, and (**c**,**f**) modified polyurethane (PU) fibrous membranes after immersed in phosphate-buffered saline (PBS) suspension of (**a**–**c**) *S. aureus*, or (**d**–**f**) *E. coli* for 4 h. Scale bar = 5 μm. (**g**) Cell viability study of *S. aureus*, and (**h**) *E. coli* as a function time in contact with control (filled square), pristine (filled circle), and modified PU fibrous membranes (filled triangle). Reproduced with permission from [153].

In parallel to the immobilization of macromolecules on the surface of fibers to promote wound healing, the ability to provide antimicrobial and antibacterial properties enables the use of electrospun fibers in most biomedical disciplines and devices. For example, the surface of the electrospun polyurethane fibers was modified by 4-vinylpyridine through UV-induced graft copolymerization [153]. The modified polyurethane (PU) fibers showed a six-fold reduction of *Staphylococcus aureus* and a three-fold reduction of *Escherichia coli* over 4 h of incubation as compared to the control (Figure 5). Others coated the surface of the alginate fibers with chitosan to provide antibacterial properties against *Staphylococcus epidermidis*, *Escherichia coli*, and various strains of *Staphylococcus aureus* [154]. Results showed that there were significant bacteria reduction rates after 6 h of contact with various lengths of the fibers (>76%), where an increasing fiber length increased bacteria reduction rate.

Overall, surface functionalization of electrospun fibers preserves the porous structure of the dressings, allowing for the exchange of oxygen and fluids. The grafted macromolecules provide additional health benefits in wound healing, and thus, making electrospun fibers the ideal candidate for advanced dressing materials.

### 5.2. Drug Eluting Stents

The application of electrospun fibers in drug-eluting stents has generated interest in the cardiovascular field in the past decade. The development of drug-eluting stents has become possible through the understanding of the biology of restenosis, controlled-release drug delivery strategies, and the use of the stent as a delivery platform (Table 3). In a study, paclitaxel-loaded chitosan core-shell fibers were used to coat a stent to provide the sustained release of paclitaxel [155]. Results suggested a cumulative release of 34% and 62% over 21 days when paclitaxel loading was 40.18 wt % and 26.19 wt %, respectively. In addition, fibers showed low cytotoxicity and good hemocompatibility against NIH/3T3 mouse embryonic fibroblasts. In another study, poly(d,l)-lactide-*co*-glycolide (PLGA) and vancomycin were electrospun into fibers and were further attached to commercial and endovascular aortic stent grafts [156]. In vitro release on vancomycin showed sustained release profiles over 30 days with 80% and 100% cumulative release of the drug for 4:1 and 6:1 (*w*/*w*) of PLGA:vancomycin fibers, respectively. In addition, the in vivo rabbit model suggested a sustained and steady release of high concentrations of vancomycin for up to eight weeks after stent implantation. Others developed bioabsorbable bifurcation stents, consisting of a double-slit tubular main body and two spiral branches, which were coated with rosuvastatin and paclitaxel-loaded electrospun PLGA fibers [157]. Results suggested that rosuvastatin and paclitaxel achieved 100% cumulative release for up to 27 days and 70 days, respectively, with the ability to reduce platelet adhesion and promote proliferation of smooth muscle cells. In general, using drug-eluting fibers to coat the interior of the vascular stents has become a trend in treating cardiovascular diseases. However, the depletion of the drug over time and the degradation of the polymeric fibers may require a second surgery to reload the drug-eluting fibers, which may not be practical for long-term care.

### 5.3. Nanofiber Scaffolds for Tissue Engineering

Many studies have utilized the surface texture of fibrous mats to provide cell stimulatory cues for the promotion of cell adhesion, orientation, migration, proliferation, differentiation, and new tissue formation [163]. These tailored fibrous mats have been widely used as scaffolding materials for applications that are related to spinal cord injuries, nerve tissue engineering, cell repair, bone tissue engineering, and etc.

Spinal cord injuries affect millions of people around the world, and it has been reported that there are approximately 10.4 to 83 new incidents per million population per year worldwide [164]. When considering the treatment of spinal cord injuries using scaffolds in tissue engineering, a newly developed peptide hydrogel scaffold from FGL, which is a peptide motif from neural cell adhesion molecule, showed the ability to self-assemble into nanofibrous morphology that promoted the migration of spinal cord-derived neural stem cells into the three-dimensional scaffold, followed by their proliferation [158]. Others showed improvements in the healing of spinal cord injuries in a rat model by using self-assembled nanofiber scaffolds to bridge the injured spinal cord [165]. Results demonstrated the up-regulation of migration of host cell and axons into the scaffolds and the growth of blood vessels, suggesting that the fiber-based scaffolds provided a suitable three-dimensional environment to promote the biological events of cells in tissue engineering. 

Due to its structural similarity to extracellular matrices, electrospun fibers have been used as substrates for neural regeneration in cell cultures. For example, a self-assembling peptide RADA16-I-BMHP1 incorporated PLGA nanofiber scaffold was developed to promote the adhesion and proliferation of rat Schwann cell [159]. In addition, the peptide blended PLGA nanofiber scaffolds significantly upregulated the expression of the cultures in Semaphorin3F (SEMA3F), neuropilin2 (NRP2), and plexin1 (PLX1) genes, suggesting the role of peptide sequence in axonal regeneration. In another work, various compositions of PCL/gelatin biocomposite scaffolds were fabricated for nerve regeneration, and the 70/30 PCL/gelatin scaffolds appeared to exhibit the most balanced properties in interaction with nerve stem cells (C17.2 cells) in culture [160]. Furthermore, the aligned fiber scaffolds improved cell proliferation over six days in the culture.

Electrospun nanofibers can also be applied to bone tissue engineering. In a study, various compositions of nano-hydroxyapatite and multi-walled carbon nanotubes were silanized and electrospun with a polyvinyl alcohol solution for fabrication of bioactive and biocompatible fiber scaffolds [166]. Results suggested a two-fold increase in the number of blood vessels when using scaffolds with nano-hydroxyapatite and multi-walled carbon nanotubes as compared to the control in a chick chorioallantoic membrane assay. Others applied calcium phosphate coating to PCL fiber scaffolds, followed by mineralization of the fibers in 10× simulated body fluid for 2 h [167]. After 7 days, the coating transformed into pure calcium deficient type B carbonate apatite with nano-crystallinity, which improved its biocompatibility, as the structure was similar to biological apatite. Further, the mineralized calcium phosphate coated PCL fibers showed an increase in wettability that can become potential cell carriers in bone tissue engineering.

**Figure 6:**
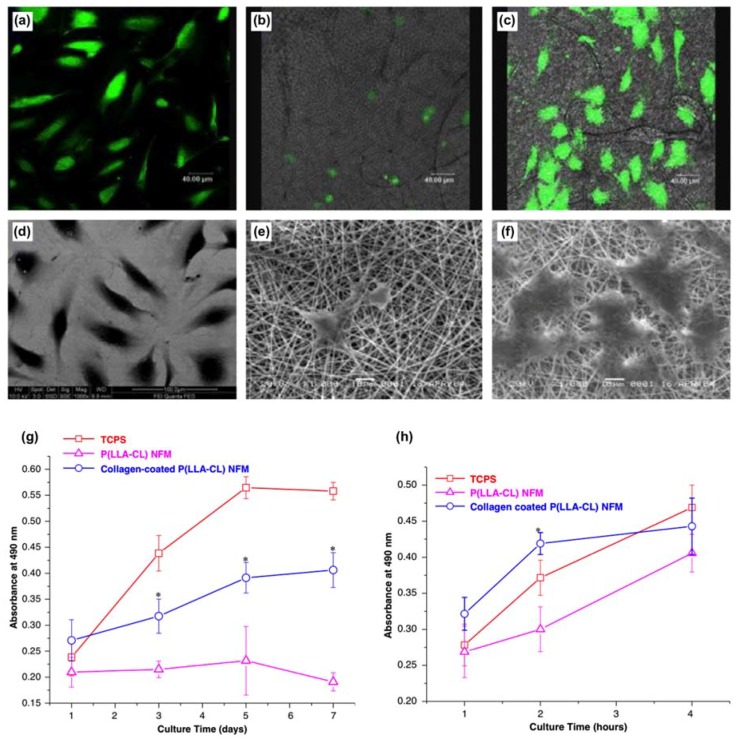
Laser scanning confocal microscopic and scanning electron microscopic images of human coronary artery endothelial cells cultured after three days on (**a**,**d**) tissue culture polystyrene plates, (**b**,**e**) poly(l-lactic acid)-*co*-polycaprolactone nanofiber meshes, and (**c**,**f**) collagen-coated poly(l-lactic acid)-*co*-polycaprolactone nanofiber meshes. (**g**) The viability of human coronary artery endothelial cells on various substrates for seven days (*n* = 3). An asterisk denotes a significance of *p* < 0.05 compared to the poly(l-lactic acid)-*co*-polycaprolactone nanofiber meshes group. (**h**) Attachment of human coronary artery endothelial cells on various substrates after four days (*n* = 3). An asterisk denotes a significance of *p* < 0.05 as compared to the poly(l-lactic acid)-*co*-polycaprolactone nanofiber meshes group. Reproduced with permission from [162].

Vascular grafts are in high demand due to the needs for engineering arterial prostheses. Electrospun fibrous scaffolds are among the most promising candidate for the application [168]. For example, the surface of the electrospun PCL nanofibers was grafted with gelatin molecules to improve their compatibility with endothelial cells for the use as a blood vessel scaffold [161]. Results suggested that gelatin grafted PCL fibers increased the spreading and proliferation of endothelial cells as compared to the blank fibers. In addition, the aligned gelatin-grafted PCL fibers showed a better ability to orient endothelial cells along the fibers when comparing to the nonwoven PCL fibers. The gelatin grafted PCL fiber scaffolds promote gene expressions in platelet-endothelial cell adhesion molecule 1 (PECAM-1), intercellular adhesion molecule 1 (ICAM-1), and vascular cell adhesion molecule 1 (VCAM-I), suggesting the potential use for surface-grafted PCL fibrous scaffolds in blood vessel tissue engineering. Others used collagen-coated poly(l-lactic acid)-*co*-polycaprolactone fiber scaffolds to attract the attachment and the phenotypic maintenance of human coronary artery endothelial cells in applications using tissue-engineered vascular grafts (Figure 6) [162]. Results suggested a three-fold increase in cell density over seven days of culture, where the expression of platelet-endothelial cell adhesion molecule 1 was preserved on collagen-coated poly(l-lactic acid)-*co*-polycaprolactone fiber scaffolds as compared to the control groups (tissue culture polystyrene plates).

Overall, fiber scaffolds have been widely used in cell cultures for various tissue engineering applications. The abilities to promote cell proliferation while maintaining cell phenotypic expression are beneficial in cell therapy and regenerative medicine.

## 6. Future Directions

**Figure 7:**
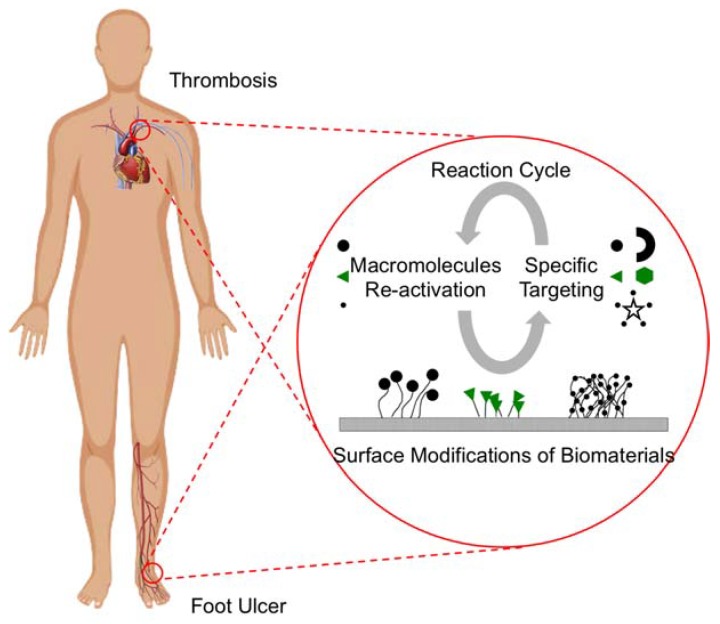
A schematic diagram of proposed future direction for biomedical devices with surface attachments of macromolecules. The macromolecules contain specific binding with target molecules and/or cells, and the macromolecules become activated again in the body allowing reattachment to the specific sites for the next functional cycle.

In light of the advances in nanotechnology, grafting of macromolecules onto the surface of polymeric coatings can create a nanostructure-enabled and/or nanotextured surface that is capable of acting as a functional device to perform the specific biomedical functions. While the various combinations of attachment methods and the types of macromolecules that are used are seemingly endless, the next breakthrough is likely on the long-term performance of these macromolecule-grafted biomaterials, which also depends on the types of diseases or pathologies they target. For example, vascular stents are required to be implanted in the body for several years and must be able to resist inflammation and thrombosis during this time period to prevent rejection, whereas foot ulcers may need weeks to months of treatment (Figure 7). As such, the design of the biomaterials and their surface modifications may be tailored for each particular disease. However, this approach will require a tremendous amount of effort since each device needs to be tailor-made for each patient.

As a result, a potential solution for future direction is to research the long-term efficacy and the effectiveness of injectable and implantable biomaterials by utilizing various "regeneration" methods for macromolecules that are attached to the surface of biomaterials. For example, macromolecules grafted onto a surface coating on vascular stents may be designed for later controlled release (by normal attrition or a triggered release) from the surface along with their clotting factors or enzyme targets to be carried away by the circulation. These combined molecules circulate in the body and they may be eliminated or separated by a secondary redox reaction. Therefore, the macromolecules are activated again for further re-attachment at specific sites on the stent while the proteins and/or enzymes are carried away by our body’s own metabolism system (Figure 7). This particular scheme can also be applied to topical dressing materials for non-healing wounds, where the replenishment of the surface macromolecules can be done via local enhancement of growth factors or by simply removing the materials to attach more macromolecules. The proof-of-principle of this concept has been demonstrated in a work to observe the redox behavior of Trolox, a vitamin E analog, in the presence of macromolecule-bound antioxidants [169]. By utilizing such a technique, the device will be able to withstand long-term use to avoid post-procedures in removing and replacing the materials. In general, biomaterials with surface nanostructures and/or nanotextures will become more popular since they exhibit a better biocompatibility to the surrounding tissues while simultaneously promoting cellular functions for therapeutic purposes.

## 7. Conclusions

In summary, surface modifications of the injectable and implantable biomaterials improve their biocompatibility and enable biomedical functions in thrombosis prevention and wound healing. In this review, we discussed various grafting methods to functionalize the surfaces of biomaterials for further attachment of macromolecules. Current research showed that nanostructure-enabled coating surfaces exhibited a particular nanotexture that acts as an active device to prevent thrombosis formation that is caused by traditional bare-metal stents. In addition, the grafting of macromolecules on fiber surfaces showed ability in the controlled release of drugs to promote wound healing. The improvement of surface functionalization enables the long-term use of injectable and implantable biomaterials with low device rejection rate. Finally, we illustrate our vision in future directions of surface modifications in biomedical devices where we emphasize the concept of ”re-activation” of these macromolecule-grafted surfaces. Our review provides an in-depth discussion of current technologies and future trends in nanostructure—enabled and macromolecule-grafted surfaces for biomedical applications.

## Figures and Tables

**Table 1:** Metal-based stents and anti-coagulation films/coatings for thrombosis applications.

Metal Stents	Films/Coatings	Methods	Functions	Ref.
Nickel-titanium (NiTi)	Polytetrafluoroethylene (PTFE)	The NiTi substrate was nano-roughened with target-ion induced plasma sputtering (TIPS) and coated with a 60% PTFE layer.	A better bonding of the PTFE film/coating to the NiTi stent for improvement in blood compatibility	[94]
Metal-stent	Phosphorylcholine	A four-beam laser interference is used to alter the metal-stent and next coated with a layered phosphorylcholine and miRNA126 coating.	Altering the surface profile of a metal stent to achieve drug delivery for coronary heart disease.	[95]
Ti6A14V alloy	Zirconium oxide	The surface of the Ti6A14V alloy is modified by implanting zirconium oxide using a plasma ion implanter.	The zirconium oxide film/coating on the Ti6A14V alloy aids wear resistance for medical implantations.	[96]
Titanium	TiO_2_ topography (TNT), (SLA), and (SLA/TNT)	Titanium implants.	Improving osseointegration and promotion of biological response to mesenchymal stem cells (MSC).	[97]
Stainless steel	(AU/NPSS)	The stainless-steel stent is encrusted with self-organized gold nanostructures.	Stainless steel/AU/NPSS is an electrochemical biosensor for measurements of dopamine levels.	[98]
Methacrylic acid (MAA)	Thermoplastic polyurethane (TPU)	MAA is grafted by argon plasma or UV light to TPU.	Enhancing blood compatibility and human bone marrow cell adhesion.	[99]
2-hydroxyethyl methacrylate (HEMA)	Thermoplastic polyurethane (TPU)	HEMA is grafted by argon plasma or UV light to TPU.	Improving biocompatibility with lower levels of thrombogenicity for the use of coatings in heart valves.	[100]
Polyallylamine	CD47	Polyallylamine bisphosphonate-modified.	Inhibition of cellular inflammatory response.	[101]

**Table 2:** Stimuli-responsive polymers for drug delivery applications.

Polymers Under a Stimuli-Response	Applications	Drug Delivery	Ref.
1. pH-sensitive polymers:			
Poly(ethylene glycol)-poly-(aspartate hydrazine adriamycin)	Anticancer drug delivery	Adriamycin	[118]
Poly(methacrylic acid-grafted-poly(ethylene glycol)	Cytotoxic effects	Proteins and peptides	[119]
	Drug delivery	Cisplatin	[120]
*N*-Acryloyl-l-phenylalanine grafted sodium alginate copolymer, *N*-isopropylacrylamide, acrylamide	Anticancer drug delivery	Imatinib mesylate	[121]
Methoxyl poly(ethylene glycol)-poly(caprolactone)-acryloyl chloride, poly(ethylene glycol) methyl ether methacrylate, and methacrylic acid	Oral drug delivery	Dexamethasone	[122]
Alginate and chemical modified carboxymethyl chitosan	Oral drug delivery	Bovine serum albumin	[123]
2. Temperature-sensitive polymers:			
*N*-Acryloyl-l-phenylalanine grafted sodium alginate copolymer, *N*-isopropylacrylamide, acrylamide	Anticancer drug delivery	Imatinib mesylate	[121]
Conjugated linoleic acid coupled with pluronic F-127	Peritoneal dissemination of gastric cancer	Docetaxel	[124]
Poly-(lactic-*co*-glycolic acid) and polyethylene glycol copolymer	Anticancer drug delivery	Chemokine stromal cell-derived factor-1α	[125]
Chitosan, collagen, α, β-glycerophosphate	Mimic extracellular microenvironment for tissue regeneration	Aid tissue regeneration	[126]
Polybenzofulvene derivative	Anticancer drug delivery	Leuprolide	[127]
3. Light-sensitive polymers:			
Region-regular poly(3-hexylthiophene) polymer	Use for triggered drug release or depolarization and hyperpolarization of the cell membrane	N/A	[128]
4. Electro-responsive polymers:			
Poly(ethyleneimine) and 1-vinylimidazole	Transdermal drug delivery	Indomethacin	[129]
5. Magnetic-sensitive polymers:			
Sodium alginate and iron oxide nanoparticles	Ocular drug delivery	Diclofenac sodium	[130]
Polyethylene glycol and 3-(trimethoxysilyl)propyl methacrylate coated magnetic nanoparticles	Cocaine recognition.	N/A	[131]
6. Multi stimuli-responsive polymers: (e.g., pH and temperature-responsive)			
*N*-isopropylacrylamide, acrylic acid	Use for drug delivery	Caffeine	[132]
*N*-isopropylacrylamide, acrylic acid, and vinyl terminated polydimethylsiloxane	Coatings on drug tablets	Indomethacin	[133]

**Table 3:** Nanostructured fibers for tissue engineering applications.

Nanostructured Fibers	Growth Factors	Performance	Ref.
Polycaprolactone (PCL) and polyethylene glycol (PEG) coaxial fibers	Fibroblast growth factor (bFGF) and epidermal growth factor (EGF)	A study inferred that 2% of EGF was released in a week from PCL-PEG shell and 30% of bFGF encapsulated in the core was released in 12 h.	[149]
Immobilized fibers	Human cathelicidin peptide LL37 (Cysk-KR12)	Antimicrobial peptide motif (Cysk-KR12) was able to maintain antibacterial properties for 3 weeks. This study concluded that Cysk-KR12 activated keratinocytes, fibroblasts, and monocytes.	[150]
Polycaprolactone (PCL)	Peptide E7Arg-Gly-Asp peptide (RGD)	This study showed that PCL/E7 attained high percentage on MSCs growth than PCL/RGD and lowered inflammatory cells.	[151]
Polycaprolactone (PCL)	Soluble eggshell membrane protein (SEP)	A study stated that SEP-grafted PCL fibers were more hydrophilic than the blank PCL fibers, which promoted human dermal fibroblasts (HDFs) growth.	[152]
Hydrogel scaffold from FGL	FGL—A peptide motif from neural cell adhesion molecule	The nanofibrous morphology supported the migration and growth of spinal cord neural stem cells into a 3-dimensional scaffold. This study attained a promising treatment for spinal cord injuries using scaffold in tissue engineering.	[158]
PLGA or poly (lactic-*co*-glycolic acid) nanofiber scaffold	Peptide (RADA16-I-BMHP1)	A study integrated PLGA to a self-assembling peptide (RADA16-I-BMHP1) to assist adhesion and growth of rat Schwann cells. The peptide/PLGA blend upregulated genes expressions in the cultures: (SEMA3F), (NRP2), and (PLX1).	[159]
Polycaprolactone (PCL)	Gelatin molecules	A study manufactured PCL/Gelatin scaffold for nerve regeneration and 70/30 PCL/Gelatin to promote nerve stem cells in culture.	[160]
Polycaprolactone (PCL)	Gelatin molecules	A study suggested that PCL/gelatin assist spreading and growth of endothelial cells compared to a blank PCL surface.	[161]
Poly (l-lactic acid)-*co*-polycaprolactone fiber scaffold	Collagen	A study derived that collagen coated poly (l-lactic acid)-*co*-polycaprolactone fiber scaffold benefit attachment of endothelial cells in tissue engineering vascular grafts.	[162]
